# Transdiagnostic neurocognitive subgroups and functional course in young people with emerging mental disorders: a cohort study

**DOI:** 10.1192/bjo.2020.12

**Published:** 2020-03-19

**Authors:** Jacob J. Crouse, Kate M. Chitty, Frank Iorfino, Joanne S. Carpenter, Django White, Alissa Nichles, Natalia Zmicerevska, Ashleigh M. Tickell, Rico S.C. Lee, Sharon L. Naismith, Elizabeth M. Scott, Jan Scott, Daniel F. Hermens, Ian B. Hickie

**Affiliations:** Brain and Mind Centre, University of Sydney, Australia; Translational Australian Clinical Toxicology (TACT) Research Group, University of Sydney, NSW, Australia; Brain and Mind Centre, University of Sydney, Australia; and InnoWell, Pty Ltd, Australia; The Black Dog Institute, University of New South Wales, Australia; Turner Institute for Brain and Mental Health, Monash University, Australia; Charles Perkins Centre, University of Sydney; and Brain and Mind Centre, University of Sydney, Australia; Notre Dame Medical School, University of Notre Dame, Australia; Academic Psychiatry, Institute of Neuroscience, Newcastle University, UK; Sunshine Coast Mind and Neuroscience Thompson Institute, University of the Sunshine Coast, Australia

**Keywords:** Social functioning, outcome studies, psychotic disorders, anxiety disorders, depressive disorders

## Abstract

**Background:**

Neurocognitive impairments robustly predict functional outcome. However, heterogeneity in neurocognition is common within diagnostic groups, and data-driven analyses reveal homogeneous neurocognitive subgroups cutting across diagnostic boundaries.

**Aims:**

To determine whether data-driven neurocognitive subgroups of young people with emerging mental disorders are associated with 3-year functional course.

**Method:**

Model-based cluster analysis was applied to neurocognitive test scores across nine domains from 629 young people accessing mental health clinics. Cluster groups were compared on demographic, clinical and substance-use measures. Mixed-effects models explored associations between cluster-group membership and socio-occupational functioning (using the Social and Occupational Functioning Assessment Scale) over 3 years, adjusted for gender, premorbid IQ, level of education, depressive, positive, negative and manic symptoms, and diagnosis of a primary psychotic disorder.

**Results:**

Cluster analysis of neurocognitive test scores derived three subgroups described as ‘normal range’ (*n* = 243, 38.6%), ‘intermediate impairment’ (*n* = 252, 40.1%), and ‘global impairment’ (*n* = 134, 21.3%). The major mental disorder categories (depressive, anxiety, bipolar, psychotic and other) were represented in each neurocognitive subgroup. The global impairment subgroup had lower functioning for 3 years of follow-up; however, neither the global impairment (*B* = 0.26, 95% CI −0.67 to 1.20; *P* = 0.581) or intermediate impairment (*B* = 0.46, 95% CI −0.26 to 1.19; *P* = 0.211) subgroups differed from the normal range subgroup in their rate of change in functioning over time.

**Conclusions:**

Neurocognitive impairment may follow a continuum of severity across the major syndrome-based mental disorders, with data-driven neurocognitive subgroups predictive of functional course. Of note, the global impairment subgroup had longstanding functional impairment despite continuing engagement with clinical services.

Mental disorders are a leading cause of functional disability worldwide.^1^ Although the adverse impacts of these disorders on work, study and relationships are experienced across the lifespan, their significance is especially negative during the formative years of adolescence and young adulthood.^1^ Birth cohort studies show that a mental disorder such as anxiety or depression during adolescence is prognostic of a range of adverse life outcomes including reduced workforce participation, academic underachievement and welfare dependence.^2–4^ As early social and economic disengagement can have long-term scarring effects on later social and health outcomes,^5^ it is vital that we improve our understanding of the barriers to social and occupational functioning in young people in the early phases of mental disorders.

## Neurocognition in mental disorders

One of the strongest predictors of social and occupational functioning in mental disorders is neurocognition. This relationship has high face validity – skills related to work, study and social interaction require an ability to learn and remember new information and flexibly shift processing across changing tasks and environments. Meta-analyses demonstrate that many individuals with depressive, bipolar and psychotic disorders have impairments of small-to-large magnitude across most measured neurocognitive domains,^6–8^ and mounting evidence shows that neurocognitive impairments limit adaptive functioning across these disorders.^9–12^ Importantly however, heterogeneity in neurocognition is common within the major mental disorders,^13^ and diagnosis-level analysis may obscure neurocognition–functioning relationships.

## Data-driven neurocognitive subgroups in mental disorders

One potential way to reframe neurocognition in mental disorders is to search for subgroups with greater homogeneity than is found in the major diagnostic groupings. To this end, data-driven statistical techniques such as cluster analysis have been used to derive neurocognitive subgroups within samples of people with schizophrenia for three decades.^14^ Data-driven studies in schizophrenia and more broadly defined psychotic disorders have typically separated patients into subgroups of global neurocognitive impairment, normal range ability and mixed or intermediate profiles.^14–17^ Recently, evidence of similar subgroups have been shown within samples of participants with depressive^18^ and bipolar disorders,^19^ and notably, across broader samples comprised of people with multiple major diagnostic groups.^20–23^ Taken together, these findings of homogeneous subgroups within diagnostic groups suggest that neurocognitive impairment may follow a continuum of severity distributed across mental disorders, with data-driven subgroups potentially representing a more useful level of analysis as regards neurocognition and associated factors.

## The current study

To date, the predictive utility of data-driven neurocognitive subgroups has not been robustly evaluated. Several studies have shown that neurocognitive subgroups within psychotic disorders have different levels of social and occupational functioning cross-sectionally,^15,17^ and one study has reported distinct courses of functioning over 6 months among neurocognitive subgroups with first-episode psychosis.^16^ Two questions with potential clinical implications remain unanswered. First: are neurocognitive subgroups associated with functional course for a greater duration than 6 months? And second: does the relationship between neurocognitive subgroups and functional course extend to broader transdiagnostic samples? Accordingly, this study aimed to determine whether data-driven neurocognitive subgroups of adolescents and young adults with emerging mental disorders are associated with distinct courses of social and occupational functioning over 3 years of contact with clinical services. Secondarily, we aimed to determine whether these subgroups differ in clinical or sociodemographic factors that may be modifiable or explain neurocognitive differences. Based on previous work,^16^ we expected that the subgroup with the greatest neurocognitive impairment would have the poorest course of functioning for at least 6 months.

## Method

### Participants

Participants were drawn from a cohort of 6743 consecutive referrals to youth mental health clinics at the Brain and Mind Centre in Sydney, Australia, who were recruited to a case register of adolescents and young adults with mood, psychotic, developmental and other mental disorders between 2004 and 2018 (‘Brain and Mind Research Institute Patient Research Register’).^24^ These clinics (for example headspace) provide youth-friendly and highly accessible early-intervention services for young people with emerging substance use and/or mental disorders, and primarily attracts young people with a range of subthreshold and threshold mental health problems (typically anxiety and mood syndromes).^24^ headspace consists of an integrated mix of primary-level services and more specialised services (for example psychiatry, drug and alcohol, occupational support), and all participants were receiving clinician-based case management and relevant social, psychological and/or medical treatments as part of standard care, which may have involved contact with a psychiatrist, psychologist, occupational therapist, support worker or admission to hospital for those whose need exceeded the capacity of the services.

### Eligibility criteria

Eligibility criteria for this study were:

- a neurocognitive assessment with no missing data across nine predetermined domains;
- aged 12 to 30 years at the time of neurocognitive assessment;
- a proforma assessment (see below) within 3 months of the neurocognitive assessment (see Iorfino et al^25^ for more detail); and
- willing and able to give informed consent (and/or parental consent was obtained).

Exclusion criteria were:

- history of neurological disease;
- medical illness known to affect neurocognitive/brain function (for example cancer, epilepsy);
- received electroconvulsive therapy in the 3 months prior to assessment;
- clinically evident intellectual disability; and/or
- insufficient understanding of the English language to allow participation in verbal assessments or testing.

### Ethics approval and informed consent

The authors assert that all procedures contributing to this work comply with the ethical standards of the relevant national and institutional committees on human experimentation and with the Helsinki Declaration of 1975, as revised in 2008. All procedures involving patients were approved by the University of Sydney Human Research Ethics Committee (project numbers: 2012/1626, 2012/1631). Written informed consent was obtained from participants aged 16 and older, and parental/guardian consent was obtained for participants younger than 16 years.

### Outcome variable (longitudinal)

A standardised clinical proforma was used to gather retrospective demographic, clinical, and functioning data from clinical case files across up to eight predetermined time points (baseline, 3 months, 6 months, 1 year, 2 years, 3 years, 4 years and 5 years). The proforma collects standardised information^25^ regarding: (a) basic demographics; (b) mental health diagnoses (based on DSM-5 criteria^26^); (c) clinical course (for example admission to hospital); (d) comorbidities (such as physical health diagnoses); and (e) functioning. Phase I of data extraction of the Optymise cohort concluded in 2018 and comprised 2767 individuals who were initially recruited to the Brain and Mind Research Institute Patient Research Register (*n* = 6743).

The outcome variable for this study was social and occupational functioning, as measured by the Social and Occupational Functioning Assessment Scale (SOFAS).^27^ The SOFAS is a 100-point scale (higher scores denoting better functioning), with instructions that the rater avoid confounding the rating of functioning with symptoms. The SOFAS is widely used and has good construct validity,^28^ interrater reliability^28^ and predictive validity.^29^

### Predictor variables (baseline)

A subset of the wider cohort participated in clinical and neurocognitive assessments between 2008 and 2015 as part of a neurobiological study. A board-certified neuropsychologist, research psychologist or supervised doctoral student administered the neurocognitive battery assessing the following domains: processing speed (Trail Making Test, part-A),^30^ cognitive flexibility (Trail Making Test, part-B),^30^ verbal learning (sum of trials 1–5 of the Rey Auditory Verbal Learning Test; RAVLT),^31^ verbal memory (20-minute delayed recall of the RAVLT),^31^ sustained attention (A’ Prime subtest of the Rapid Visual Information Processing Test),^32^ set-shifting (Intra-Extra Dimensional Set Shift),^32^ visuospatial memory (Paired Associates Learning Task),^32^ working memory (Spatial Span Task),^32^ and verbal fluency (Controlled Oral Word Association Test, letters).^33^ Premorbid intellectual functioning (premorbid IQ) was estimated using word-reading tests; the Wide Range Achievement Test (fourth edition)^34^ was used for participants younger than 16 years and the Wechsler Test of Adult Reading^35^ was used for participants older than 16 years. Neurocognitive test scores were standardised to age- and gender-matched norms (*z*-scores) using established criteria, as described previously.^36,37^ To avoid derivation of small, independent subgroups influenced by extreme scores,^23^ *z*-scores beyond 5.0 or −5.0 were winsorised to 5.0 or −5.0, depending on the direction.

The 24-item Brief Psychiatric Rating Scale (BPRS)^38^ measured symptom type and severity, with four dimensions derived (depressive, positive, negative and manic). The 10-item Kessler Psychological Distress scale (K10)^39^ measured perceived severity of psychological distress. Age at onset of psychiatric symptoms was self-reported, and duration of illness was estimated by subtracting age at onset from age at baseline assessment. The World Health Organization's Alcohol, Substance, and Smoking Involvement Screening Test version 2.0 (WHO-ASSIST 2.0)^40^ measured lifetime and recent (past 3 months) substance use. We added a question to item one (lifetime use) to estimate age of first use: ‘If yes, at what age did you first use?’. The 10-item Alcohol Use Disorders Identification Test (AUDIT)^41^ assessed severity of alcohol use.

As neurocognition was the key baseline predictor in this study, the nearest proforma assessment within 3 months of the neurocognitive assessment was selected as the participants’ baseline proforma time point (*T*_1_), and subsequent proforma time points were accordingly recoded. As we allowed a 3-month interval between the neurocognitive and proforma assessments, the 3-month proforma time point was excluded from analysis. The 4- and 5-year time points were also excluded from analysis as sample attrition exceeded 80%.

### Statistical analysis

Analyses were performed using R statistical software, version 3.4.2 (R Foundation).^42^

#### Model-based cluster analysis

The *mclust* package,^43^ version 5.4.1, was used to derive subgroups of participants based on neurocognitive *z*-scores across nine domains. The ‘Mclust’ function uses mixture modelling via expectation-maximisation algorithms to iteratively fit a variety of covariance structures to the data, comparing Bayesian Information Criterion (BIC) values for each model to select the optimal data structure (a larger BIC indicates stronger evidence for a corresponding model). The ‘Mclust’ function fits 14 covariance structures across nine components (clusters) as default. Once an optimal solution was selected (based on model fit and parsimony), cluster groups were compared on sociodemographic and clinical factors using one-way analysis of variance for continuous variables and χ^2^-tests for categorical variables.

#### Mixed-effects modelling

Linear mixed-effects models were built using the *nlme* package, version 3.1-137,^44^ with missing follow-up data handled using maximum-likelihood estimation. The outcome variable was participants’ SOFAS scores at each time point. SOFAS scores for all available time points were used, and participants could contribute one or multiple scores over time.

Analyses were conducted sequentially. First, we built an unconditional model with random intercepts and no predictor variables, positing a linear trajectory in SOFAS over time across the sample. Next, we determined whether the model fit could be improved by fitting random slopes, with goodness-of-fit compared using the likelihood ratio test (LRT) statistic, which expresses how many times more likely the data are under one model relative to another. We then built a conditional model, testing interindividual differences in functioning at baseline and the rate of change in functioning over time as a function of several predetermined factors. A ‘time’ variable represented the time point of each SOFAS score and was coded numerically. To avoid listwise deletion of participants with missing predictors, we imputed missing predictor data with the sample mean before modelling (no more than 8% of data were missing for any predictor; see supplementary Table 1 available at https://doi.org/10.1192/bjo.2020.12). Normality of residuals was visually inspected using Q–Q plots, with an approximate normal distribution evident. Multicollinearity was evaluated using the variation inflation factor (VIF), with no predictor observed to have a VIF over 2. Model coefficients (*B*) are presenting alongside 95% confidence intervals, test statistic and parameter-specific *P*-values.

## Results

### Participant characteristics

A total of 2767 participants from the wider Optymise cohort had an available proforma assessment. Of these, 629 participants met all eligibility criteria (see supplementary Fig. 1 for participant flow). At baseline, there were 629 participants and of these 350 were female (55.6%) and 279 were male (44.4%), with a median age of 20 (interquartile range 6). More than 90% of the sample were aged 12–25 years.

The majority of participants presented with a primary mood or anxiety disorder (428/629, 68.0%). Numbers and proportions of each primary diagnostic group were: depressive disorders (244/629, 38.8%), anxiety disorders (96/629, 15.3%), bipolar and related disorders (88/629, 14.0%), schizophrenia spectrum and other psychotic disorders (82/629, 13.0%), neurodevelopmental disorders (37/629, 5.9%) disruptive, impulse-control and conduct disorders (20/629, 3.2%), substance-use and addictive disorders (12/629, 1.9%), trauma- and stressor-related disorders (12/629, 1.9%), obsessive–compulsive and related disorders (10/629, 1.6%), personality disorders (6/629, 1.0%) and feeding and eating disorders (4/629, 0.6%). There was a total of 18 participants who had no diagnosis or an uncertain diagnosis (2.9%).

### Cluster solution

The results of the cluster analysis across the nine neurocognitive tests indicated that the optimal model was a seven-cluster solution with an ellipsoidal, equal orientation covariance structure and the second-best model was an eight-cluster solution with the same covariance structure (BICs for all solutions are presented in supplementary Table 2). However, the third best model was a three-cluster solution with the same covariance structure and a similar BIC (best: seven-cluster BIC = −16 147.2; second best: eight-cluster BIC = −16 233.29; third best: three-cluster BIC = −161 241.5). As the three-cluster solution was more parsimonious and largely capitulated the largest cluster groups from the other solutions, we selected the three-cluster solution.

### Demographic and clinical characteristics of the three cluster groups

**Fig. 1:**
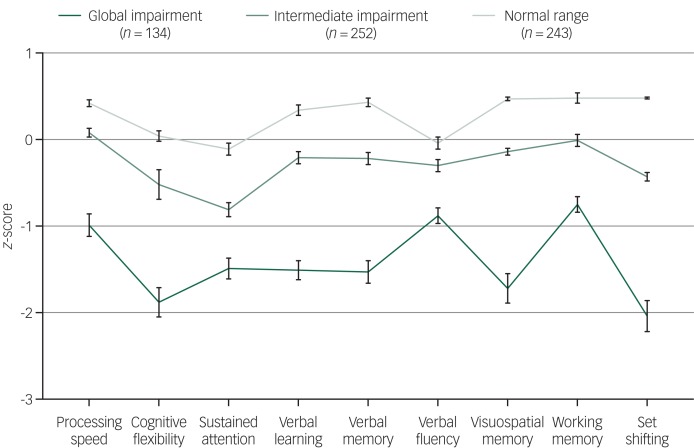
Baseline neurocognitive profiles (*z*-scores and s.e.) for the three cluster groups of 629 adolescents and young adults with emerging mental disorders.

**Fig. 2:**
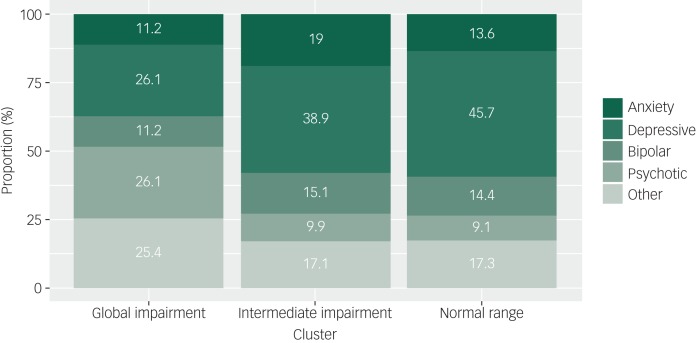
Proportions of major diagnostic groups allocated to each neurocognitive cluster group.

The three neurocognitive cluster groups were best described as ‘global impairment’ (*n* = 134; 21% total sample), ‘intermediate impairment’ (*n* = 252; 40% total sample), and ‘normal range’ (*n* = 243; 39% total sample) (Fig. 1). As shown in Table 1, cluster-group differences were observed for gender, level of education, premorbid IQ, baseline SOFAS, level of negative and positive symptom severity, and daily tobacco use (pairwise comparisons correcting for multiple comparisons are in Table 1). No significant differences were observed for age, level of depressive or manic symptom severity, psychological distress, self-reported age of psychiatric symptoms onset, estimated duration of illness, or any other substance use parameter. Of note, primary diagnostic groups were distributed across the three cluster groups, albeit unevenly (Table 2 and Fig. 2).

**Table 1:** Demographic, clinical, and substance use characteristics of three neurocognitive cluster groups

Characteristic	Cluster 1, global impairment (*n* = 134)		Cluster 2, intermediate impairment (*n* = 252)		Cluster 3, normal range (*n* = 243)		Statistics			Effect size (Cohen's *d*)		
							d.f.	*F* or χ^2^	*P*	1 *v.* 2	2 *v.* 3	1 *v.* 3
Demographic												
Age, years: mean (s.d.)	19.9	(3.7)	19.8	(4.2)	20.1	(4.1)	2, 626	0.27	0.765	–	–	–
Gender (female), *n* (%)	51/134	(38)	145/252	(58)	154/243	(63)	2	23.04	<0.001	–	–	–
Education, years: mean (s.d.)	11.4	(2.4)	11.6	(2.5)	12.2	(2.5)	2, 612	4.96	0.007	0.08	0.24*	0.33*
Premorbid IQ, mean (s.d.)	97.5	(10.6)	101.7	(9.2)	105.5	(8.9)	2, 589	30.50	<0.001	0.42***	0.42***	0.82***
SOFAS, mean (s.d.)	55.9	(9.8)	59.8	(9.6)	62.8	(9.4)	2, 626	22.96	<0.001	0.40***	0.32**	0.72***
Clinical status												
BPRS depressive, mean (s.d.)	13.3	(4.9)	13.5	(5.0)	13.8	(5.4)	2, 576	0.53	0.588	–	–	–
BPRS negative, mean (s.d.)	8.3	(3.3)	6.9	(2.6)	7.0	(2.7)	2, 575	10.68	<0.001	0.47***	0.04	0.43***
BPRS positive, mean (s.d.)	11.7	(4.7)	10.8	(3.8)	10.0	(2.9)	2, 577	8.24	<0.001	0.21	0.24*	0.44***
BPRS mania, mean (s.d.)	9.6	(2.9)	9.6	(3.4)	9.3	(3.1)	2, 583	0.68	0.507	–	–	–
K10, mean (s.d.)	28.0	(9.6)	27.6	(8.4)	27.3	(8.1)	2, 551	0.27	0.765	–	–	–
Age at onset, mean (s.d.)	15.8	(4.5)	14.8	(4.3)	15.0	(4.2)	2, 462	1.72	0.180	–	–	–
Illness duration, mean (s.d.)	4.4	(3.7)	5.3	(4.2)	4.9	(3.8)	2, 462	1.51	0.222			
Substance use												
Alcohol, AFU: mean (s.d.)	14.9	(2.2)	14.8	(2.6)	14.8	(2.7)	1, 434	0.09	0.916	–	–	–
Tobacco, AFU: mean (s.d.)	15.1	(2.8)	15.2	(2.5)	15.3	(2.9)	2, 325	0.12	0.883	–	–	–
Cannabis, AFU: mean (s.d.)	15.7	(2.6)	15.7	(3.2)	15.8	(2.9)	2, 304	0.05	0.947	–	–	–
ATS, AFU: mean (s.d.)	17.4	(2.7)	16.9	(4.0)	16.3	(4.8)	2, 190	0.85	0.431	–	–	–
AUDIT (total), mean (s.d.)	6.4	(8.7)	7.1	(8.2)	6.1	(6.8)	2, 540	0.85	0.427	–	–	–
Daily tobacco, *n* (%)	34/97	(35)	41/194	(21)	39/194	(20)	2	9.05	0.011	–	–	–
Weekly + cannabis, *n* (%)	13/96	(14)	21/192	(11)	23/196	(12)	2	0.42	0.811	–	–	–
Monthly + ATS,^a^ *n* (%)	8/97	(8)	9/194	(5)	8/197	(4)	–	2.43	0.304	–	–	–

SOFAS, Social and Occupational Functioning Assessment Scale; BPRS, Brief Psychiatric Rating Scale; K10, Kessler Psychological Distress Scale (10-item); AFU, age first use; ATS, Amphetamine-type stimulant; AUDIT, Alcohol Use Disorders Identification Test.

a. Fisher's exact test.

***P < 0.001; **P < 0.01; *P < 0.05 (Tukey's honest significant difference *post hoc* comparisons).

**Table 2:** Numbers and proportions (%) of each major diagnostic group within each cluster group

Diagnostic group	*n* (%)		
	Global impairment (*n* = 134)	Intermediate impairment (*n* = 252)	Normal range (*n* = 243)
Anxiety disorder	15 (11.2)	48 (19.0)	33 (13.6)
Depressive disorder	35 (26.1)	98 (38.9)	111 (45.7)
Bipolar disorder	15 (11.2)	38 (15.1)	35 (14.4)
Psychotic disorder	35 (26.1)	25 (9.9)	22 (9.1)
Other disorders	34 (25.4)^a^	43 (17.1)^b^	42 (17.3)^c^

a. Post-traumatic stress disorder (*n* = 3); adjustment disorder (*n* = 2); substance-use disorder (*n* = 4); obsessive-compulsive disorder (*n* = 2); autism spectrum disorder (*n* = 4); attention-deficit hyperactivity disorder (*n* = 3); unspecified neurodevelopmental disorder (*n* = 1); borderline personality disorder (*n* = 1); unspecified personality disorder (*n* = 1); conduct disorder (*n* = 1); oppositional defiant disorder (*n* = 5); unspecified disruptive, impulse-control, and conduct disorder (*n* = 3); uncertain/no diagnosis (*n* = 4)

b. Post-traumatic stress disorder (*n* = 1); acute stress disorder (*n* = 1); unspecified trauma- and stress-related disorder (*n* = 2); substance-use disorder (*n* = 4); obsessive-compulsive disorder (*n* = 4); autism spectrum disorder (*n* = 6); attention-deficit hyperactivity disorder (*n* = 9); unspecified neurodevelopmental disorder (*n* = 1); borderline personality disorder (*n* = 3); conduct disorder (*n* = 1); oppositional defiant disorder (*n* = 2); unspecified disruptive, impulse-control, and conduct disorder (*n* = 4); bulimia (*n* = 2); uncertain/no diagnosis (*n* = 3).

c. Post-traumatic stress disorder (*n* = 2); unspecified trauma- and stress-related disorder (*n* = 1) substance-use disorder (*n* = 4); obsessive-compulsive disorder (*n* = 4); autism spectrum disorder (*n* = 4); attention-deficit hyperactivity disorder (*n* = 9); borderline personality disorder (*n* = 1); conduct disorder (*n* = 1); oppositional defiant disorder (*n* = 2); unspecified disruptive, impulse-control, and conduct disorder (*n* = 1); bulimia (*n* = 1); unspecified eating disorder (*n* = 1); uncertain/no diagnosis (*n* = 11).

Neurocognitive profiles of the three cluster groups are presented in Fig. 1. As expected, cluster-group differences were observed for all neurocognitive domains. Cluster one (*n* = 134) had global impairment across all domains, with *z*-scores 1–2 s.d. below the norm for cognitive flexibility (−1.88), sustained attention (−1.49), verbal learning (−1.51), verbal memory (−1.53), visuospatial memory (−1.72) and set-shifting (−2.04), and z-scores 0.5–1 s.d. below the norm for processing speed (−0.99), verbal fluency (−0.88) and working memory (−0.75).

Cluster two (*n* = 252) had intermediate impairment, with all domains falling within normal limits (i.e. −1.0 to 1.0 s.d.), with only cognitive flexibility (−0.52) and sustained attention (−0.81) falling below −0.5 s.d. Cluster three (*n* = 243) had a normal range profile, with performance 0–0.5 s.d. above the norm for all domains except sustained attention (−0.11) and verbal fluency (−0.04). Importantly, cluster-group differences in neurocognitive scores remained statistically significant when adjusting for estimated premorbid IQ (supplementary Table 3).

### Associations between neurocognitive subgroups and functioning at baseline and over time

#### Unconditional model

We first constructed an unconditional model (i.e. no predictors) with random intercepts. We next included the fixed relationship between SOFAS and ‘time’ with a linear term, which was significant and indicated that SOFAS scores increased over time across all participants (*B* = 0.55, 95% CI 0.29–0.81, *P* < 0.001). Next, slopes were randomly varied across participants. This random slopes and random intercepts model fit the data substantially better than the random intercepts and fixed slopes model (LRT = 74.64, *P* < 0.001).

#### Modelling functioning at baseline and over time: unadjusted associations

Next, we modelled relationships between all predictor variables and variation in baseline SOFAS, and between cluster-group membership and the rate of change in SOFAS over time (with the normal range cluster group serving as the reference). As shown in Table 3, the unadjusted models showed significant associations between all predictor variables and baseline SOFAS. Lower baseline functioning was associated with membership in the global impairment cluster group (*B* = −6.59, 95% CI −8.50 to −4.68, *P* < 0.001), membership in the intermediate cluster group (*B* = −2.49, 95% CI −4.07 to −0.90, *P* = 0.002), male gender (*B* = −3.24, 95% CI −4.70 to −1.77, *P* < 0.001), lower premorbid IQ (*B* = 0.20, 95% CI 0.13 to 0.28, *P* < 0.001), fewer years of education (*B* = 0.80, 95% CI 0.50 to 1.09, *P* < 0.001) and greater level of depressive (*B* = −0.38, 95% CI −0.53 to −0.23, *P* < 0.001), positive (*B* = −0.74, 95% CI −0.94 to −0.55, *P* < 0.001), negative (*B* = −1.01, 95% CI −1.27 to −0.75, *P* < 0.001) and manic symptom severity (*B* = −0.36, 95% CI −0.59 to −0.12, *P* = 0.003). There was no significant difference in the rate of change in SOFAS over time for the intermediate impairment cluster group compared with the normal range cluster group (*B* = 0.27, 95% CI −0.18 to 0.71, *P* = 0.236); however, members of the global impairment cluster group had a lesser rate of SOFAS improvement over time compared with the normal range cluster group (*B* = −0.85, 95% CI −1.50 to −0.21, *P* = 0.010).

**Table 3:** Unadjusted and adjusted linear mixed-effects models (*n* = 629) examining associations between Social and Occupational Functioning Assessment Scale; intercept (i.e. baseline) and slope (i.e. longitudinal change) and neurocognitive clusters, sociodemographics, and symptom typology and severity

	Unadjusted				Adjusted			
	Coefficient	95% CI	*t*	*P*	Coefficient	95% CI	*t*	*P*
Intercept	60.53	59.79 to 61.26	161.54	<0.001	56.73	48.51 to 64.96	13.48	<0.001
Time	0.53	0.20 to 0.86	3.13	0.002	0.27	−0.25 to 0.79	1.01	0.311
Clusters								
Global impairment	−6.59^a^	−8.50 to −4.68	−6.77	<0.001	−4.18^a^	−6.57 to −1.80	−3.43	<0.001
Intermediate impairment	−2.49^a^	−4.07 to −0.90	−3.07	0.002	−2.31^a^	−4.21 to −0.41	−2.38	0.018
Sociodemographics								
Gender (male)	−3.24	−4.70 to −1.77	−4.33	<0.001	−1.96	−3.35 to −0.56	−2.73	0.006
Premorbid IQ	0.20	0.13 to 0.28	5.33	<0.001	0.11	0.04 to 0.19	2.98	0.003
Education (years)	0.80	0.50 to 1.09	5.30	<0.001	0.53	0.25 to 0.81	3.67	<0.001
Symptom severity								
BPRS depressive	−0.38	−0.53 to −0.23	−5.08	<0.001	−0.27	−0.42 to −0.12	−3.45	<0.001
BPRS positive	−0.74	−0.94 to −0.55	−7.49	<0.001	−0.34	−0.56 to −0.13	−3.16	0.002
BPRS negative	−1.01	−1.27 to −0.75	−7.63	<0.001	−0.60	−0.86 to −0.33	−4.43	<0.001
BPRS manic	−0.36	−0.59 to −0.12	−3.00	0.003	−0.07	−0.29 to 0.16	−0.59	0.555
Associations with rate of change								
Time × global impairment	−0.85	−1.50 to −0.21	−2.59	0.010	0.26^a^	−0.67 to 1.20	0.55	0.581
Time × intermediate impairment	0.27	−0.18 to 0.71	1.19	0.236	0.46^a^	−0.26 to 1.19	1.25	0.211

BPRS, Brief Psychiatric Rating Scale.

a. Normal range cluster group represents the reference category.

#### Modelling functioning at baseline and over time: adjusted associations

**Fig. 3:**
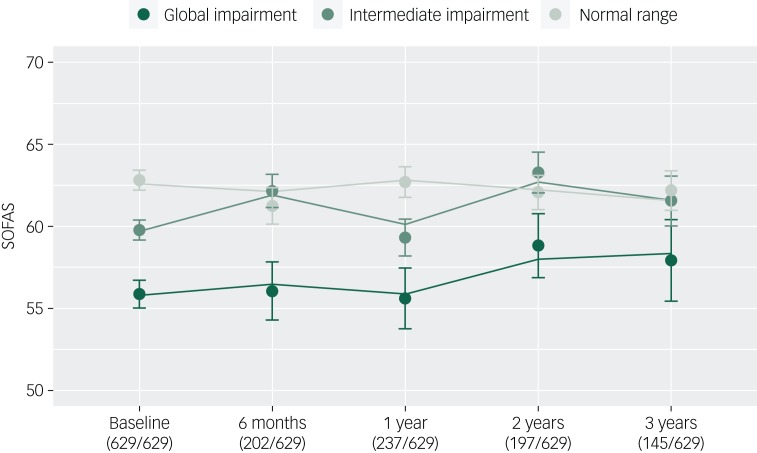
Functional trajectories of three neurocognitive clusters of young people (*n* = 629) with emerging mental disorders over 3 years of contact with clinical services (lines, fitted model; filled circles, observed data; bars, s.e.).

Finally, we examined whether the neurocognitive cluster groups would be associated with variation in baseline SOFAS and SOFAS change over time after statistical adjustment for sociodemographic and symptom variables. As shown in Table 3, lower baseline functioning was again associated with membership in the global impairment cluster group (*B* = −4.18, 95% CI −6.57 to −1.80, *P* < 0.001) and the intermediate cluster group (*B* = −2.31, 95% CI −4.21 to −0.41, *P* = 0.018) (relative to the normal range cluster), as well as male gender (*B* = −1.96, 95% CI −3.35 to −0.56, *P* < 0.006), lower premorbid IQ (*B* = 0.11, 95% CI 0.04 to 0.19, *P* = 0.003), fewer years of education (*B* = 0.53, 95% CI 0.25 to 0.81, *P* < 0.001), and greater level of depressive (*B* = −0.27, 95% CI −0.42 to −0.12, *P* < 0.001), positive (*B* = −0.34, 95% CI −0.56 to −0.13, *P* = 0.002) and negative symptom severity (*B* = −0.60, 95% CI −0.86 to −0.33, *P* < 0.001). Neither the global impairment (*B* = 0.26, 95% CI −0.67 to 1.20, *P* = 0.581) nor the intermediate cluster groups (*B* = 0.46, 95% CI −0.26 to 1.19, *P* = 0.211) differed from the normal range cluster group in their rate of change in SOFAS over time. The trajectories of functioning of the three cluster groups are presented in Fig. 3.

Proportions of participants with an assessment at each time point are presented. SOFAS, Social and Occupational Functioning Assessment Scale.

#### Sensitivity analysis

To evaluate whether associations between the global impairment cluster group and functioning were in part driven by a greater proportion of psychotic disorders in this subgroup, we included a dichotomous variable representing the presence or absence of a primary psychotic disorder at baseline. Although there was a significant relationship between functioning and having a psychotic disorder (*B* = −4.08, 95% CI −5.95 to −2.21, *P* < 0.001), all other associations remained statistically significant (supplementary Table 4).

## Discussion

### Principal findings

This study reports the longer-term course of social and occupational functioning of a large clinical cohort of adolescents and young adults accessing youth mental health services. We demonstrate for the first time that data-driven neurocognitive subgroups are predictive of functional course for up to 3 years, with a global impairment subgroup following the poorest course of functioning independent of gender, premorbid IQ, level of education, level of symptom severity and presence of a primary psychotic disorder. Notably, all major diagnostic groups were represented in each subgroup (Fig. 2). Taken together, these findings suggest neurocognitive impairment may be distributed along a continuum of severity across syndrome-based major mental disorders and is a robust and transdiagnostic predictor of functional course.

### Neurocognitive subgroups cut across major syndrome-based diagnostic groups

Our observation that primary diagnostic groups were distributed across the three cluster groups is consistent with previous work in schizophrenia and bipolar disorder^20^ and in a transdiagnostic in-patient sample.^21^ In the current study, around one-quarter of the global impairment cluster had a primary depressive disorder and another quarter had a primary bipolar or anxiety disorder. Notably, more than half of the participants with a primary psychotic disorder were allocated to the normal range or intermediate subgroups (supplementary Table 5), highlighting that within-diagnosis neurocognitive heterogeneity may be obscured by diagnosis-level comparisons, which tend to report a gradient of worst impairment in psychotic disorders, followed by bipolar and depressive disorders.^13,20^ However, consistent with other transdiagnostic – or cross-diagnostic – studies,^17,20,21^ participants with psychotic disorders were overrepresented in the global impairment subgroup. Biological factors such as brain abnormalities or genetic risk for neurocognitive impairment may be important factors for such individuals with global impairment, as reported in several studies of neurocognitively impaired subgroups with schizophrenia.^45–48^ These factors may also be relevant for bipolar and other non-psychotic disorders, especially given the degree of shared genetic risk across the major mental disorders.^49–52^

### Sociodemographic and clinical differences between neurocognitive subgroups

Several important factors differed between the neurocognitive subgroups. First, the global impairment subgroup had lower premorbid IQ and an overrepresentation of males, providing some evidence in support of a neurodevelopmental component in this group. Second, there were group differences in positive and negative symptoms, which might be explained by the greater proportion of psychotic disorders in the global impairment subgroup relative to the normal range subgroup (26.1% *v.* 9.1%), or alternatively, by shared precursors to neurocognitive impairments and positive and negative symptoms. Finally, tobacco use was more common in the global impairment subgroup, which might be explained by higher rates of tobacco use in individuals with psychosis^53^ or acute self-medication of neurocognitive impairments, although support for the latter is equivocal.^54^

### Strengths of the study

Several strengths of this study are worth mentioning. The cohort was a large group of young people accessing transdiagnostic youth mental health services, and the naturalistic design gives insight into real-world patterns of functioning over time, which may be generalisable to similar transdiagnostic youth mental health services that are emerging around the world in Australia, the UK, Ireland, Canada, Denmark, Asia and the USA.^55^ Second, multiple ratings of functioning allowed us to model the rate of change in functioning over time, building on many previous reports examining only one or two follow-up time points. Third, this is one of the largest studies of its kind, with most cross-sectional neurocognitive cluster studies totalling fewer than 200 participants. Fourth, we extend previous findings of an association between neurocognitive cluster group and functional course from 6 months^16^ to 3 years, and show broader implications across mood, anxiety, psychotic and other disorders.

### Limitations

Several limitations are worth mentioning. First, studies in schizophrenia consistently report mediation of the path from neurocognition to functional outcome by several factors that were unmeasured here, including social cognition and intrinsic motivation^56^; they are likely relevant beyond schizophrenia. Second, we relied on a single neurocognitive assessment and cannot evaluate the stability of our neurocognitive subgroups over time. Third, sample attrition (Fig. 3) may have biased model estimates; however, differences between participants lost to follow-up and retained were small (supplementary Table 6). Fourth, there were differences in neurocognitive test scores across the major diagnostic groups (supplementary Table 7), and it is possible that a subgroup of participants with severe psychotic disorders may have influenced our findings; however we also adjusted our models for the presence of a psychotic disorder (supplementary Table 4). Fifth, there was some evidence of bimodal distributions for cognitive flexibility and set-shifting in the global impairment cluster (supplementary Fig. 2a–i), which may have influenced the mean severity of this cluster group. However, model residuals were approximately normally distributed, meeting a key assumption of the mixed-effects framework. Sixth, the wide age-range of the participants may mean that age-related neurocognitive test heterogeneity may have influenced our results. However, there were no significant differences between cluster groups in age (*P* = 0.765) and differences in neurocognitive test scores between participants below 18 years (*n* = 191) versus those aged 18 years and over (*n* = 438) were small (supplementary Table 8). Finally, individuals in the global impairment subgroup were more likely to be using antipsychotic medication, which is likely related to the overrepresentation of psychotic disorders in this group (supplementary Table 9). However, rates of missing medication data did not allow us to model medication as a covariate.

### Implications and future directions

Taken together, our results support the strong association between neurocognitive ability and social and occupational functioning among young people with emerging mental disorders, with novel transdiagnostic and longitudinal implications. Longitudinal studies before and after illness onset are needed to identify unique and/or shared genetic or neurodevelopmental pathways to neurocognitive impairment, that may speculatively evolve independently of later syndrome-based diagnostic group. These studies will be important to determine whether observed subgroups represent biologically meaningful, ‘natural kinds’ of groupings,^57^ or instead represent segments of a neurocognitive continuum distributed throughout the population. Moreover, future studies should utilise machine-learning approaches to better select variables to be used in clustering algorithms (for example Dwyer et al^58^), and to broaden outcome variables to model relationships between data-driven subgroups and other clinical and functional outcomes (for example clinical stage transition, admission to hospital), which may assist in planning of personalised interventions.^59^

## Data Availability

De-identified data may be made available from the corresponding author upon reasonable request.

## Supplementary material

For supplementary material accompanying this paper visit http://dx.doi.org/10.1192/bjo.2020.12. click here to view supplementary material
