# Supplementary material for: Transdiagnostic neurocognitive subgroups and functional course in young people with emerging mental disorders: a cohort study
Source: BJPsych Open. 2020 Mar 19;6(2):e31. doi: 10.1192/bjo.2020.12 (PMC7176869; doi:10.1192/bjo.2020.12)
Supplement: Supplementary file 1 [file S2056472420000125sup001.zip › Crouse_BJPsychOpen-09-0145_R1_Supplementary_Table_1.docx]

**Supplementary Table 1. Number and proportion of participants with missing data for each baseline predictor variable.**

| **Variable** | **N (%)** |
| --- | --- |
| Gender | 0 (0%) |
| Education | 14 (2.2) |
| Premorbid IQ | 37 (5.9) |
| BPRS depressive | 50 (7.9) |
| BPRS negative | 51(8.1) |
| BPRS positive | 49 (7.8) |
| BPRS mania | 43 (6.8) |
| TMT-A | 0 (0.0) |
| TMT-B | 0 (0.0) |
| RAVLT-sum | 0 (0.0) |
| RAVLT-delayed | 0 (0.0) |
| RVP | 0 (0.0) |
| PAL | 0 (0.0) |
| SSP | 0 (0.0) |
| COWAT | 0 (0.0) |
| IED | 0 (0.0) |

*Note*: BPRS = Brief Psychiatric Rating Scale; TMT-A = Trail Making Test, part-A; TMT-B = Trail Making Test, part-B; RAVLT-sum = Rey Auditory Verbal Learning Test, sum of trials 1-5 of the Rey Auditory Verbal Learning Test; RAVLT-delayed = Rey Auditory Verbal Learning Test, 20-minute delayed recall; RVP = A’ Prime subtest of the Rapid Visual Information Processing Test; PAL = Paired Associates Learning Task; SSP = Spatial Span Task; COWAT = Controlled Oral Word Association Test, letters; IED = Intra-Extra Dimensional Set Shift
