# Supplementary material for: Transdiagnostic neurocognitive subgroups and functional course in young people with emerging mental disorders: a cohort study
Source: BJPsych Open. 2020 Mar 19;6(2):e31. doi: 10.1192/bjo.2020.12 (PMC7176869; doi:10.1192/bjo.2020.12)
Supplement: Supplementary file 1 [file S2056472420000125sup001.zip › Crouse_BJPsychOpen-09-0145_R1_Supplementary_Table_2.docx]

**Supplementary Table 2. Bayesian Information Criterion for 14 parameterized covariance structures across 9 components (3 best fitting models are in bold and shaded in green).**

| **Components** | **EII** | **VII** | **EEI** | **VEI** | **EVI** | **VVI** | **EEE** | **EVE** | **VEE** | **VVE** | **EEV** | **VEV** | **EVV** | **VVV** |
| --- | --- | --- | --- | --- | --- | --- | --- | --- | --- | --- | --- | --- | --- | --- |
| **1** | -19098.57 | -19098.57 | -19014.96 | -19014.96 | -19014.96 | -19014.96 | -17415.94 | -17415.94 | -17415.94 | -17415.94 | -17415.94 | -17415.94 | -17415.94 | -17415.94 |
| **2** | -17936.22 | -17411.76 | -17886.87 | -17392.24 | -17628.23 | -17053.03 | -17063.24 | -16919.47 | -16805.76 | -16583.80 | -17015.25 | -16585.32 | -17018.22 | -16628.17 |
| **3** | -17737.57 | -17110.00 | -17540.84 | -17072.58 | -17474.50 | -16630.22 | -17199.85 | -16753.74 | -16718.00 | **-16241.53** | -17045.23 | -16483.72 | -16999.33 | -16690.92 |
| **4** | -17476.46 | -17049.49 | -17462.73 | -17011.25 | -17242.82 | -16523.39 | -17258.18 | -16595.26 | -16689.88 | -16292.44 | -17020.29 | -16569.11 | -17101.53 | -16616.10 |
| **5** | -17375.96 | -16995.04 | -17391.30 | -16948.19 | -17196.01 | -16522.26 | -16675.75 | -16802.28 | -16727.32 | -16372.18 | -17190.16 | -16704.70 | -17322.99 | 16832.96 |
| **6** | -17254.65 | -16984.42 | -17316.02 | -16957.63 | -17067.36 | -16472.57 | -16931.93 | -16440.39 | -16752.29 | -16334.25 | -17218.02 | -16928.02 | -17342.50 | -16979.66 |
| **7** | -17225.60 | -16917.86 | -17220.29 | -16898.91 | -16850.48 | -16406.15 | -16983.53 | -16450.98 | -16441.09 | **-16147.21** | -17151.09 | -17032.96 | -17450.84 | -17257.06 |
| **8** | -17250.97 | -16888.80 | -17212.98 | -16824.84 | -16861.37 | -16467.27 | -17026.87 | -16558.19 | -16461.97 | **-16233.29** | -17350.90 | -17299.91 | -17706.88 | -17478.03 |
| **9** | -17199.41 | -16891.17 | -17252.87 | -16815.03 | -16874.22 | -16531.06 | -17063.08 | -16509.30 | -16482.83 | -16321.45 | -17583.33 | -17482.23 | -17952.65 | -17700.03 |

*Note*: EII = “spherical, equal volume”; VII = “spherical, unequal volume”; EEI = “diagonal, equal volume and shape”; VEI = “diagonal, varying volume, equal shape”; EVI = “diagonal, equal volume, varying shape”; VVI = “diagonal, varying volume and shape”; EEE = “ellipsoidal, equal volume, shape, and orientation”; EVE = “ellipsoidal, equal volume and orientation”; VEE = “ellipsoidal, equal shape and orientation”; VVE = “ellipsoidal, equal orientation”; EEV = “ellipsoidal, equal volume and equal shape”; VEV = “ellipsoidal, equal shape”; EVV = “ellipsoidal, equal volume”; VVV = “ellipsoidal, varying volume, shape, and orientation”.
