# Supplementary material for: Transdiagnostic neurocognitive subgroups and functional course in young people with emerging mental disorders: a cohort study
Source: BJPsych Open. 2020 Mar 19;6(2):e31. doi: 10.1192/bjo.2020.12 (PMC7176869; doi:10.1192/bjo.2020.12)
Supplement: Supplementary file 1 [file S2056472420000125sup001.zip › Crouse_BJPsychOpen-09-0145_R1_Supplementary_Table_3.docx]

**Supplementary Table 3. Neurocognitive performance (z-scores) of three neurocognitive cluster-groups.**

|  | **Cluster 1**  **(N=134)**  **“Global impairment”** | | **Cluster 2**  **(N=252)**  **“Intermediate impairment”** | | **Cluster 3**  **(N=243)**  **“Normal-**  **range”** | | **ANOVA *F (p)***  **[df = 2, 626]** | | **Effect size, d** | | |
| --- | --- | --- | --- | --- | --- | --- | --- | --- | --- | --- | --- |
| **Domain** | **M** | **SD** | **M** | **SD** | **M** | **SD** |  |  | **1 *v.* 2** | **2 *v.* 3** | **1 *v.* 3** |
| Processing Speed | -0.99 | 1.55 | 0.08 | 0.80 | 0.42 | 0.68 | 93.44 | *** ^a^ | 0.87 | 0.46 | 1.18 |
| Cognitive Flexibility | -1.88 | 1.93 | -0.52 | 1.35 | 0.04 | 0.99 | 84.42 | *** ^a^ | 0.82 | 0.47 | 1.25 |
| Sustained Attention | -1.49 | 1.37 | -0.81 | 1.28 | -0.11 | 1.07 | 57.36 | *** ^a^ | 0.51 | 0.59 | 1.12 |
| Verbal Learning | -1.51 | 1.30 | -0.21 | 1.10 | 0.34 | 0.92 | 127.00 | *** ^a^ | 1.08 | 0.54 | 1.64 |
| Verbal Memory | -1.53 | 1.52 | -0.22 | 1.13 | 0.43 | 0.81 | 132.30 | *** ^a^ | 0.98 | 0.66 | 1.61 |
| Verbal Fluency | -0.88 | 1.05 | -0.30 | 1.04 | -0.04 | 1.16 | 25.68 | *** ^a^ | 0.56 | 0.24 | 0.76 |
| Visuospatial Memory | -1.72 | 1.98 | -0.14 | 0.68 | 0.47 | 0.25 | 203.10 | *** ^a^ | 1.07 | 1.19 | 1.55 |
| Working Memory | -0.75 | 1.10 | -0.01 | 1.13 | 0.48 | 0.92 | 59.55 | *** ^a^ | 0.66 | 0.48 | 1.21 |
| Set-Shifting | -2.04 | 2.10 | -0.43 | 0.86 | 0.48 | 0.17 | 221.20 | *** ^a^ | 1.00 | 1.47 | 1.69 |

*Note* *** = p<0.001; ** = p<0.01; * = p<0.05; df = degrees of freedom. ^a^ remained significant after controlling for estimated premorbid IQ.
