# Supplementary material for: Transdiagnostic neurocognitive subgroups and functional course in young people with emerging mental disorders: a cohort study
Source: BJPsych Open. 2020 Mar 19;6(2):e31. doi: 10.1192/bjo.2020.12 (PMC7176869; doi:10.1192/bjo.2020.12)
Supplement: Supplementary file 1 [file S2056472420000125sup001.zip › Crouse_BJPsychOpen-09-0145_R1_Supplementary_Table_4.docx]

**Supplementary Table 4. Adjusted linear mixed effects model (N=629) examining associations between SOFAS intercept (i.e. baseline) and slope (i.e. longitudinal change) and neurocognitive clusters, socio-demographics, symptom typology and severity, and primary psychotic disorder diagnosis.**

|  | **Unadjusted** | | | **Adjusted** | | | | |
| --- | --- | --- | --- | --- | --- | --- | --- | --- |
|  | **Coefficient [95% CI]**  **SE** | **t**  **CI** | **p** | | **Coefficient [95% CI]**  **SE** | **t**  **CI** | **p** | |
| **Intercept** | 60.53 [59.79, 61.26] | 161.54 | <0.001 | | 56.23 [48.09, 64.37] | 13.49 | <0.001 | |
| **Time** | 0.53 [0.20, 0.86] | 3.13 | 0.002 | | 0.25 [-0.27, 0.77] | 0.92 | 0.355 | |
| **Cluster-group** |  | | |  | | | | |
| Global impairment | -6.59 [-8.50, -4.68] ^a^ | -6.77 | <0.001 | | -3.87 [-6.23, -1.51] ^a^ | -3.203 | 0.001 | |
| Intermediate impairment | -2.49 [-4.07, -0.90] ^a^ | -3.07 | 0.002 | | -2.41 [-4.29, -0.54] ^a^ | -2.51 | 0.012 | |
| **Socio-demographics** |  | | |  | | | | |
| Gender (male) | -3.24 [-4.70, -1.77] | -4.33 | <0.001 | | -1.65 [-3.04, -0.26] | -2.32 | 0.021 | |
| Predicted IQ | 0.20 [0.13, 0.28] | 5.33 | <0.001 | | 0.10 [0.03, 0.18] | 2.79 | 0.006 | |
| Education (years) | 0.80 [0.50, 1.09] | 5.30 | <0.001 | | 0.62 [0.34, 0.90] | 4.34 | <0.001 | |
| **Symptom severity** |  | | |  | | | | |
| BPRS Depressive | -0.38 [-0.53, -0.23] | -5.08 | <0.001 | | -0.34 [-0.49, -0.18] | -4.29 | <0.001 | |
| BPRS Positive | -0.74 [-0.94, -0.55] | -7.49 | <0.001 | | -0.25 [-0.46, -0.03] | -2.23 | 0.026 | |
| BPRS Negative | -1.01 [-1.27, -0.75] | -7.63 | <0.001 | | -0.48 [-0.75, -0.22] | -3.53 | <0.001 | |
| BPRS Mania | -0.36 [-0.59, -0.12] | -3.00 | 0.003 | | -0.11 [-0.33, 0.12] | -0.95 | 0.341 | |
| **Diagnosis** |  |  |  | |  |  |  | |
| Psychotic disorder | -5.37 [-7.23, -3.51] | -5.67 | <0.001 | | -4.08 [-5.95, -2.21] | -4.26 | <0.001 | |
| **Associations with rate of change** |  | | |  | | | | |
| Time x Global impairment | -0.85 [-1.50, -0.21] | -2.59 | 0.010 | | 0.28 [-0.65, 1.21] ^a^ | 0.59 | | 0.557 |
| Time x Intermediate impairment | 0.27 [-0.18, 0.71] | 1.19 | 0.236 | | 0.56 [-0.16, 1.29] ^a^ | 1.52 | | 0.128 |

*Note*: BPRS = Brief Psychiatric Rating Scale; 95% CI = 95% Confidence Interval; ^a^ Normal range cluster-group represents the reference category
