# Supplementary material for: Transdiagnostic neurocognitive subgroups and functional course in young people with emerging mental disorders: a cohort study
Source: BJPsych Open. 2020 Mar 19;6(2):e31. doi: 10.1192/bjo.2020.12 (PMC7176869; doi:10.1192/bjo.2020.12)
Supplement: Supplementary file 1 [file S2056472420000125sup001.zip › Crouse_BJPsychOpen-09-0145_R1_Supplementary_Table_5.docx]

**Supplementary Table 5. Numbers and proportions (%) of each cluster-group within each primary diagnostic group.**

|  | **Anxiety disorder**  **(N=96)** | **Depressive disorder**  **(N=244)** | **Bipolar disorder**  **(N=88)** | **Psychotic disorder**  **(N=82)** | **Other disorder**  **(N=119)** |
| --- | --- | --- | --- | --- | --- |
| Global impairment | 15 (15.6) | 35 (14.3) | 15 (17.0) | 35 (42.7) | 34 (28.6) |
| Intermediate impairment | 48 (0.50) | 98 (40.2) | 38 (43.2) | 25 (30.5) | 43 (36.1) |
| Normal range | 33 (34.4) | 111 (45.5) | 35 (39.8) | 22 (26.8) | 42 (35.3) |

*Note:* Other disorder includes: neurodevelopmental disorders; disruptive, impulse-control and conduct disorders; substance use and addictive disorders; trauma- and stressor-related disorders; obsessive-compulsive and related disorders; personality disorders; feeding and eating disorders; and no or uncertain diagnosis.
