# Supplementary material for: Transdiagnostic neurocognitive subgroups and functional course in young people with emerging mental disorders: a cohort study
Source: BJPsych Open. 2020 Mar 19;6(2):e31. doi: 10.1192/bjo.2020.12 (PMC7176869; doi:10.1192/bjo.2020.12)
Supplement: Supplementary file 1 [file S2056472420000125sup001.zip › Crouse_BJPsychOpen-09-0145_R1_Supplementary_Table_6.docx]

**Supplementary Table 6. Group-differences between participants retained at 3-year follow-up (N=145) compared to participants lost-to-follow-up (N=484).** *Note*: statistically significant differences (p<0.05) are in bold.

|  | **Lost-to-follow-up**  **(n=484)** | **Retained**  **(n=145)** | **Statistics** | |
| --- | --- | --- | --- | --- |
|  | **N or %** | **N or %** | **t or χ^2^** | **p** |
| **Age, years** | **20.17** | **19.21** | **2.72** | **0.007** |
| Gender, female | 263/484 (54%) | 87/145 (60%) | 1.23 | 0.268 |
| Education, years | 11.85 | 11.48 | 1.69 | 0.093 |
| Premorbid IQ | 101.98 | 103.42 | -1.63 | 0.104 |
| K10 | 27.48 | 27.74 | -0.32 | 0.753 |
| **SOFAS** | **60.58** | **58.54** | **2.31** | **0.022** |
| **BPRS, depressive** | **13.35** | **14.39** | **-2.20** | **0.029** |
| BPRS, negative | 7.23 | 7.36 | -0.51 | 0.608 |
| BPRS, positive | 10.66 | 10.81 | -0.45 | 0.656 |
| BPRS, mania | 9.49 | 9.30 | 0.73 | 0.465 |
| Processing Speed | -0.03 | 0.04 | -0.73 | 0.467 |
| Cognitive Flexibility | -0.63 | -0.46 | -1.11 | 0.269 |
| Sustained Attention | -0.68 | -0.69 | 0.07 | 0.947 |
| **Verbal Learning** | **-0.36** | **0.00** | **-2.97** | **0.003** |
| Verbal Memory | **-0.32** | **0.01** | **-2.68** | **0.008** |
| Verbal Fluency | -0.31 | -0.37 | 0.55 | 0.580 |
| Set-Shifting | -0.38 | 0.15 | -0.88 | 0.381 |
| Visuospatial Memory | -0.24 | -0.25 | 0.06 | 0.951 |
| Working Memory | 0.01 | 0.05 | -0.35 | 0.728 |

*Note*: K10 = Kessler Psychological Distress Scale (10-item); SOFAS = Social and Occupational Functioning Assessment Scale; BPRS = Brief Psychiatric Rating Scale
