# Supplementary material for: Transdiagnostic neurocognitive subgroups and functional course in young people with emerging mental disorders: a cohort study
Source: BJPsych Open. 2020 Mar 19;6(2):e31. doi: 10.1192/bjo.2020.12 (PMC7176869; doi:10.1192/bjo.2020.12)
Supplement: Supplementary file 1 [file S2056472420000125sup001.zip › Crouse_BJPsychOpen-09-0145_R1_Supplementary_Table_7.docx]

|  | **Anxiety**  **disorder**  **(N=96)** | | **Depressive**  **disorder**  **(N=244)** | | **Bipolar disorder**  **(N=88)** | | **Psychotic disorder**  **(N=82)** | | **Other**  **disorders**  **(N=119)** | |
| --- | --- | --- | --- | --- | --- | --- | --- | --- | --- | --- |
| **Neurocognitive test** | **M** | **SD** | **M** | **SD** | **M** | **SD** | **M** | **SD** | **M** | **SD** |
| Processing Speed | 0.01 | 1.14 | 0.06 | 1.01 | 0.10 | 1.19 | -0.29 | 1.05 | -0.09 | 1.23 |
| Cognitive Flexibility | -0.52 | 1.57 | -0.39 | 1.41 | -0.34 | 1.44 | -1.16 | 1.75 | -0.86 | 1.65 |
| Sustained Attention | -0.58 | 1.34 | -0.43 | 1.25 | -0.61 | 1.32 | -1.04 | 1.31 | -1.12 | 1.37 |
| Verbal Learning | -0.11 | 1.14 | -0.03 | 1.17 | -0.21 | 1.22 | -1.06 | 1.31 | -0.44 | 1.42 |
| Verbal Memory | -0.03 | 1.23 | -0.04 | 1.25 | -0.19 | 1.31 | -1.06 | 1.41 | -0.33 | 1.35 |
| Verbal Fluency | -0.42 | 0.97 | -0.20 | 1.10 | -0.01 | 1.11 | -0.60 | 1.03 | -0.54 | 1.30 |
| Visuospatial Memory | -0.41 | 1.43 | 0.01 | 0.99 | -0.27 | 1.15 | -0.96 | 1.78 | -0.10 | 1.32 |
| Working Memory | -0.05 | 1.27 | 0.19 | 1.12 | 0.03 | 1.21 | -0.42 | 0.97 | 0.05 | 1.07 |
| Set-Shifting | -0.19 | 1.08 | -0.18 | 1.19 | -0.27 | 1.36 | -1.00 | 1.87 | -0.82 | 1.75 |

**Supplementary Table 7. Neurocognitive test performance by primary diagnostic group.**
