# Supplementary material for: Transdiagnostic neurocognitive subgroups and functional course in young people with emerging mental disorders: a cohort study
Source: BJPsych Open. 2020 Mar 19;6(2):e31. doi: 10.1192/bjo.2020.12 (PMC7176869; doi:10.1192/bjo.2020.12)
Supplement: Supplementary file 1 [file S2056472420000125sup001.zip › Crouse_BJPsychOpen-09-0145_R1_Supplementary_Table_8.docx]

|  | **12- to 17-years**  **(N=191)** | | **18- to 30-years**  **(N=438)** | | **Test**  **statistic** | | **Effect**  **size** |
| --- | --- | --- | --- | --- | --- | --- | --- |
| **Neurocognitive test** | **M** | **SD** | **M** | **SD** | **t** | **p** | **Cohen’s d** |
| Processing Speed | -0.10 | 1.17 | 0.02 | 1.08 | -1.92 | 0.055 | 0.11 |
| Cognitive Flexibility | -0.64 | 1.36 | -0.57 | 1.63 | 1.11 | 0.267 | 0.06 |
| **Sustained Attention** | **-0.95** | **1.25** | **-0.57** | **1.35** | **-3.14** | **0.002** | **-0.17** |
| Verbal Learning | -0.19 | 1.40 | -0.32 | 1.22 | 1.71 | 0.088 | 0.10 |
| **Verbal Memory** | **-0.11** | **1.29** | **-0.31** | **1.35** | **2.37** | **0.018** | **0.12** |
| Verbal Fluency | -0.40 | 1.20 | -0.29 | 1.10 | 0.21 | 0.832 | 0.01 |
| **Visuospatial Memory** | **0.11** | **1.08** | **-0.39** | **1.36** | **7.21** | **<0.001** | **0.36** |
| **Working Memory** | **0.20** | **1.05** | **-0.05** | **1.17** | **5.30** | **<0.001** | **0.28** |
| Set-Shifting | -0.38 | 1.35 | -0.44 | 1.50 | 1.73 | 0.085 | 0.09 |

**Supplementary Table 8. Neurocognitive test scores among participants aged 12 to 17 years and 18 to 30 years.** *Note*: statistically significant differences (p<0.05) are in bold.
