# Supplementary material for: Transdiagnostic neurocognitive subgroups and functional course in young people with emerging mental disorders: a cohort study
Source: BJPsych Open. 2020 Mar 19;6(2):e31. doi: 10.1192/bjo.2020.12 (PMC7176869; doi:10.1192/bjo.2020.12)
Supplement: Supplementary file 1 [file S2056472420000125sup001.zip › Crouse_BJPsychOpen-09-0145_R1_Supplementary_Table_9.docx]

**Supplementary Table 9. Medication usage across three neurocognitive cluster-groups.** *Note*: statistically significant differences (p<0.05) are in bold.

|  | **Gross impairment**  **(N=134)** ^a^ | **Intermediate**  **impairment**  **(N=252)** ^b^ | **Normal**  **range**  **(N=243)** ^c^ | **Statistical test** | |
| --- | --- | --- | --- | --- | --- |
|  | **N (%)** | **N (%)** | **N (%)** | **χ^2^** | **p** |
| **Any antidepressant** | 55 (47.0) | 110 (50.0) | 122 (55.2) | 2.36 | 0.308 |
| **Any antipsychotic** | **64 (54.7)** | **82 (37.3)** | **63 (28.5)** | **22.41** | **<0.001** |
| **Any mood stabiliser** | 27 (23.1) | 37 (16.8) | 34 (15.4) | 3.27 | 0.195 |
| **Any other** ^d^ | 10 (8.5) | 26 (11.8) | 28 (12.7) | 1.32 | 0.516 |
| **Combination** | 47 (40.2) | 74 (33.6) | 74 (33.5) | 1.78 | 0.411 |
| **No medication** | 24 (17.9) | 54 (24.5) | 63 (28.5) | 2.69 | 0.261 |

*Note*:

^a^ Medication data available for 117/134 (87.3%)

^b^ Medication data available for 220/252 (87.3%)

^c^ Medication data available for 221/243 (90.9%)

^d^ Includes stimulants, sedatives, and benzodiazepines
