# Supplementary material for: Transdiagnostic neurocognitive subgroups and functional course in young people with emerging mental disorders: a cohort study
Source: BJPsych Open. 2020 Mar 19;6(2):e31. doi: 10.1192/bjo.2020.12 (PMC7176869; doi:10.1192/bjo.2020.12)
Supplement: Supplementary file 1 [file S2056472420000125sup001.zip › Crouse_BJPsychOpen-09-0145_Supplementary_Figure_1.docx]

**Supplementary Fig 1. Participant flow diagram.**

*Did not meet eligibility criteria* (N=2000)

- No neurocognitive assessment
- Missing neurocognitive data
- No pro forma assessment within 3-months of neurocognitive assessment
- Older than 30 years

Excluded from Phase 1 of *Optymise* Cohort (N=3976)

- Case files unavailable or incomplete
- Not allocated to Phase 1 of data extraction

Recruited to *Brain and Mind Research Institute Patient Research Register* (N=6743)

Included in Phase I *Optymise* *Cohort* (N=2767)

Included in final analysis

(N=767)
