# Supplementary material for: Transdiagnostic neurocognitive subgroups and functional course in young people with emerging mental disorders: a cohort study
Source: BJPsych Open. 2020 Mar 19;6(2):e31. doi: 10.1192/bjo.2020.12 (PMC7176869; doi:10.1192/bjo.2020.12)
Supplement: Supplementary file 1 [file S2056472420000125sup001.zip › Crouse_BJPsychOpen-09-0145_Supplementary_Figure_2.docx]

**Supplementary Figure 2A-I. Distributions of neurocognitive test performance among three cluster-groups across nine neurocognitive domains.**

**2A) Processing speed	2B) Cognitive flexibility**

**C) Sustained attention	D) Verbal learning**

**E) Verbal memory	F) Visuospatial memory**

**G) Set shifting	H) Working memory**

**I) Verbal fluency**
